# Evaluation of the Allplex™ GI-Helminth(I) Assay, the first marketed multiplex PCR for helminth diagnosis

**DOI:** 10.1051/parasite/2021034

**Published:** 2021-04-02

**Authors:** Brice Autier, Jean-Pierre Gangneux, Florence Robert-Gangneux

**Affiliations:** 1 Université de Rennes, CHU Rennes, Inserm, EHESP, Irset (Institut de Recherche en Santé Environnement Travail), UMRS 1085 35000 Rennes France

**Keywords:** *Taenia* spp., *Strongyloides* spp., Hookworms, *Enterobius vermicularis*, *Ascaris* spp.

## Abstract

Molecular biology has been gaining more importance in parasitology. Recently, a commercial multiplex PCR assay detecting helminths was marketed: the Allplex™ GI-Helminth(I) Assay. It targets *Ancylostoma* spp., *Ascaris* spp., *Enterobius vermicularis*, *Hymenolepis* spp., *Necator americanus*, *Strongyloides* spp., *Taenia* spp. and *Trichuris trichiura*, but also the two most common microsporidia genera in human health, i.e. *Enterocytozoon* spp. and *Encephalitozoon* spp. This study aimed to evaluate and compare the Allplex™ GI-Helminth(I) Assay to classical diagnostic methods, based on a cohort of 110 stool samples positive for helminths (microscopy) or for microsporidia (PCR). Samples were stored at −80 °C until analysis by the Allplex™ GI-Helminth(I) Assay. False-negatives were re-tested with bead-beating pretreatment. Without mechanical lysis, concordance and agreement between microscopy and Allplex™ GI-Helminth(I) Assay ranged from 91% to 100% and from 0.15 to 1.00, respectively depending on the target. Concordance was perfect for *Taenia* spp. (*n* = 5) and microsporidia (*n* = 10). False-negative results were observed in 54% (6/13), 34% (4/11) and 20% (7/35) of cases, for hookworms, *E. vermicularis* and *Strongyloides* spp. detection, respectively. For these targets, pretreatment improved the results, but only slightly. *Trichuris trichiura* detection was critically low without pretreatment, as only 9% (1/11) of the samples were positive, but detection reached 91% (10/11) with bead-beating pretreatment. Mechanical lysis was also needed for *Ascaris* spp. and *Hymenolepis* spp. to reduce false-negative results from 1/8 to 1/21, respectively, to none for both. Overall, with an optimized extraction process, the Allplex™ GI-Helminth(I) Assay allows the detection of numerous parasites with roughly equivalent performance to that of microscopy, except for hookworms.

## Abstract (fr)

La biologie moléculaire a maintenant une place importante en parasitologie. Le kit Allplex™ GI-Helminth(I) Assay est le premier panel multiplex commercialisé détectant des helminthes : *Ancylostoma* spp., *Ascaris* spp., *Enterobius vermicularis*, *Hymenolepis* spp., *Necator americanus*, *Strongyloides* spp., *Taenia* spp. et *Trichuris trichiura*, mais également les deux genres de Microsporidies les plus fréquents en santé humaine, *Enterocytozoon* spp. et *Encephalitozoon* spp. Cette étude a comparé la PCR Allplex™ GI-Helminth(I) Assay aux techniques diagnostiques usuelles, sur une banque préservée à −80 °C, comprenant 110 échantillons de selles positifs à helminthes (microscopie) ou à microsporidies (PCR). Les faux négatifs ont été retestés après prétraitement par broyage en billes. Sans lyse mécanique, la concordance et l’accord entre la microscopie et le test Allplex™ GI-Helminth(I) Assay variaient respectivement de 91 % à 100 % et de 0,15 à 1,00, selon la cible. La concordance était parfaite pour *Taenia* spp. (*n* = 5) et les microsporidies (*n* = 10). Des faux négatifs ont été observés pour la détection des ankylostomes, *E. vermicularis* et *Strongyloides* spp. à des taux respectifs de 54 % (6/13), 34 % (4/11) et 20 % (7/35). Pour ces cibles, le prétraitement a peu amélioré les résultats. La détection de *T. trichiura* était défectueuse sans prétraitement, avec 9 % (1/11) de positifs, mais a atteint 91 % (10/11) après prétraitement par broyage en billes. La lyse mécanique était également nécessaire pour *Ascaris* spp. et *Hymenolepis* spp. pour réduire les faux négatifs de 1/8 et 1/21, respectivement, à aucun pour les deux. Au total, avec une optimisation de l’étape d’extraction, le test Allplex™ GI-Helminth(I) Assay permet la détection de nombreux parasites avec des performances proches de celles de la microscopie, excepté pour les ankylostomes.

## Background

For more than a century, the diagnosis of intestinal helminthiasis has relied on microscopic examination of stool samples [23]. The microscopy-based methods developed for diagnostic purposes, initially based on the concentrations of parasite eggs or larvae, and then improved by staining techniques, have not yet been outclassed in their balance of sensitivity, specificity, and cost-effectiveness. However, they have several drawbacks: they are time-consuming, they need repeated stool sampling and trained operators, and several concentration methods should be combined to detect a wide spectrum of parasites and avoid false-negative results. For some helminths, serological techniques with high sensitivity have been developed (e.g. for strongyloidiasis [7]), but cross-reactions limit their use in endemic areas and long-lasting positivity prevents their use for follow-up purposes [21]. Usually, serological techniques are most useful for tissular helminthiases, or when larvae or egg detection in feces is difficult.

In other domains of clinical microbiology, namely bacteriology and virology, molecular tools have become increasingly popular, as efficient multiplex assays allow fast, reproducible, and sensitive detection of numerous pathogens. Until recently, the use of such techniques for the detection of intestinal parasites was limited by technical issues: stool samples are rich in PCR inhibitors, and helminth DNA is protected by the shell of the eggs. In the 2000s, the first in-house molecular methods for helminths detection in human stool samples were developed [19, 24, 25], closely followed by their multiplexing [4, 22]. Recently, the first commercial multiplex PCR assay was put on the market, the Allplex™ GI-Helminth(I) Assay (GIPH) assay (Seegene Inc., Seoul, South Korea), which is coupled to an automated DNA extraction device, MICROLAB^®^ STARlet (Hamilton Company, Reno, NV, USA).

This study aimed at comparing the Allplex™ GIPH assay to classical methods on a collection of samples positive for the pathogens targeted by the assay, i.e. *Ancylostoma* spp., *Ascaris* spp., *Enterobius vermicularis*, *Hymenolepis* spp., *Necator americanus*, *Strongyloides* spp., *Taenia* spp., *Trichuris trichiura*, and microsporidia (*Enterocytozoon* spp., *Encephalitozoon* spp.).

We also tested the DNA extracts obtained with the MICROLAB^®^ STARlet with an in-house PCR technique targeting *Schistosoma mansoni*, to verify whether the quality of DNA extraction was suitable to detect this major helminth not targeted by the Allplex™ GIPH assay.

## Materials and methods

### Biological samples

In all, 110 stool samples from 85 patients were selected from a collection consisting of samples prospectively analyzed from 2016 to 2020 at the Laboratory of Parasitology of the Rennes University Hospital in the framework of routine diagnosis, as previously described [1, 2]. At reception, an aliquot was immediately stored at −80 °C without addition of preservative, allowing the future selection of positive samples. Routine helminth diagnosis relied on microscopic techniques, and microsporidia diagnosis relied on molecular biology, as detailed below. For the present study, the following stool samples positive by classical methods were selected: 8 positive for *Ascaris* spp., 11 positive for *E. vermicularis*, 13 positive for hookworms (2 for *Ancylostoma duodenale*, 6 for *N. americanus*, 5 not identified to the species level), 21 positive for *Hymenolepis nana*, 35 positive for *Strongyloides stercoralis*, 5 positive for *Taenia saginata/asiatica*, 11 positive for *T. trichiura*, 9 positive for *Enterocytozoon bieneusi*, and 1 positive for *Encephalitozoon intestinalis*. All samples were part of diagnostic investigations and no patient was previously treated for an intestinal parasitic infection.

We also selected 17 additional stool samples positive for *S. mansoni* eggs by microscopy for our complementary evaluation.

### Parasitological diagnosis

After collection, all stool samples were rapidly analyzed by microscopic examination which consisted in a direct wet mount and two concentration methods (among merthiolate–iodine–formalin, Thébault’s, Bailenger’s and Willis’ concentration methods), depending on the clinical context. If the clinical or epidemiological context indicated strongyloidiasis, a Baermann sedimentation technique and/or a 10-day Harada-Mori filter paper culture were also performed. For hookworms, species-level identification was based on morphological characteristics of the filariform larvae obtained by Harada-Mori culture, when possible. Species-level identification of the *Taenia* spp. eggs was performed by microscopic examination of proglottids emitted by the patients.

Concentration methods were performed on 1 g of stool sample, and operators examined the whole concentration pellet (several slides, if needed). Eggs were quantified as “Rare”, “Few”, “Several”, “Numerous”, or “Many” if the pellet contained fewer than 5, 5–9, 10–14, 15–24, or more than 24 eggs, respectively. This made it possible to roughly estimate the fecal egg count, expressed in eggs per gram of stools (epg). This could not be done for *S. stercoralis* larvae, as the Harada-Mori culture mimics the external life cycle of the parasite, including the reproduction of free-living adults and generation of new rhabditoid larvae. However, larvae were quantified using the same quantification scale.

### Detection of microsporidia

Diagnosis of microsporidia was based on previously described molecular methods [8, 16]. Stool samples were suspended in phosphate buffered saline (PBS) and 100 μL of supernatant was added to 900 μL of ASL buffer (Qiagen, Courtaboeuf, France). After 5 min incubation at room temperature, 200 μL of supernatant was incubated for 10 min at 70 °C before DNA extraction with the EZ1^®^ Advanced XL device (Qiagen, Courtaboeuf, France) and the EZ1^®^ DSP Virus Kit (Qiagen, Courtaboeuf, France). An exogenous DNA, DiaControlDNA™ (Diagenode Diagnostics, Liège, Belgium), was added during extraction and amplified by qPCR to assess the presence of PCR inhibitors. All DNA extracts were amplified undiluted and diluted to 1:10, using the previously described primers, probe, and cycling conditions [8, 16]. Reaction mix included TaqMan^®^ Universal PCR Master Mix reagent (Applied Biosystems France, Villebon-sur-Yvette, France) and amplification was done with the Applied Biosystems StepOnePlus™ system (Applied Biosystems France, Villebon-sur-Yvette, France). Not all patients were initially screened for microsporidia, but only those with combination of diarrhea and immunodeficiency.

### Allplex™ GI – Helminth (I) assay

Allplex™ GIPH assays were used following the recommendations of the manufacturer. Samples were thawed, and approximately 160 mg of stool was suspended in 2 mL Cary-Blair medium, using the provided swab (FecalSwab™, Copan Diagnostics Inc., Murrieta, CA, USA). After a vortex mixing step, the suspensions were incubated for 10 min at room temperature, then centrifuged for 10 min at 2000 *g*. The centrifuged suspensions were then processed by the MICROLAB^®^ STARlet device (Hamilton Company, Reno, NV, USA) with the STARMag 96 Universal Cartridge reagent (Seegene Inc., Seoul, South Korea) for DNA extraction from 200 μL of supernatant. The MICROLAB^®^ STARlet device also set up the PCR mix and the DNA extracts into 96-well plates before their amplification using a CFX96 device (Bio-Rad, Marnes-la-Coquette, France). The resulting PCR curves were analyzed with the Seegene Viewer^®^ software. Recommended controls (positive and negative controls, internal control) allowed the assessment of each series. All false-negative results of the Allplex™ GIPH were reanalyzed after a bead-beating lysis step of the whole FecalSwab™ stool suspension, using a MagNA Lyser Green Beads tube (Roche Diagnostics, Meylan, France) and the MagNA Lyser system (Roche Diagnostics, Meylan, France) for 35 s at full speed. After bead-beating, suspensions were centrifuged for 10 min at 2000 *g* before processing by the MICROLAB^®^ STARlet device, as described above.

### *Schistosoma* in-house PCR

As the target panel is not designed to detect *Schistosoma* spp., which is a frequent helminth in migrant patients, we further evaluated whether the DNA extracts obtained with the MICROLAB^®^ STARlet device were suitable for *Schistosoma* detection with our in-house *S. mansoni* PCR method [9].

First, we performed an Allplex™ GIPH assay on 17 stool samples positive for *S. mansoni* eggs by microscopy. After DNA extraction with the MICROLAB^®^ STARlet, DNA extracts were immediately collected and stored at −20 °C until amplification using the previously described primers, probe, and cycling conditions [9] with the TaqMan^®^ Universal PCR Master Mix reagent and the Applied Biosystems StepOnePlus™ device. As for microsporidia, DNA extracts were amplified plain and diluted to 1:10.

### Statistical analysis

Concordance and agreement between PCR and microscopy were evaluated using the proportions of concordance and Cohen’s *κ* coefficient [14, 26]. Sensitivity of each target was calculated as relative sensitivity by comparison to the routine technique. Proportions were compared using Fisher’s exact test.

## Results

### Allplex™ GI-Helminth(I) Assay versus microscopy

For all targets classically detected by microscopy, concordance and agreement between microscopy and Allplex™ GIPH assay ranged from 91% to 100% and from 0.15 to 1.00, respectively (Table 1). Agreement was perfect (*κ* = 1.00) for *Taenia* spp. or almost perfect (*κ* between 0.81 and 1.00) for *Ascaris* spp. and *Hymenolepis* spp. For the latter, a sample positive by microscopy with few *H. nana* eggs (5–9 epg) was not detected by the Allplex™ GIPH assay, while two other samples from the same patient, negative by microscopy, turned out to be positive by multiplex PCR. For *Ascaris* spp., a sample with <5 epg detected by microscopy was negative by PCR. Compared to microscopy, sensitivities for these targets ranged from 88% to 100% (Table 2). Agreement was considered substantial (*κ* between 0.61 and 0.80) between both methods for *E. vermicularis* and *S. stercoralis* detection, with *κ* coefficients close to the upper limit (*κ* from 0.76 to 0.80), leading to sensitivities of 64% and 80%, compared to microscopy, respectively. Among the false-negative results of *E. vermicularis* PCR, three samples had <5, 10–14, and 15–25 epg, respectively quantified by microscopy, and one sample contained one adult. For *Strongyloides* spp., 7 samples positive by microscopy, with rare (*n* = 5), several (*n* = 1) and numerous (*n* = 1) larvae, were negative with the Allplex™ GIPH assay, and 2 samples were negative by microscopy and positive with the molecular assay. One was obtained from a patient previously diagnosed as infected, and the other from a traveler who was diagnosed with hookworm eggs. Finally, the poorest agreement rates were observed for the overall results for hookworms (moderate agreement, *κ* between 0.41 and 0.60), and for *T. trichiura* (Table 1), with only 6 out of 13 samples and 1 out of 11 samples detected by PCR, respectively. For *N. americanus*, 4 of 6 samples positive by microscopy were detected by PCR, for the 2 remaining ones, the fecal egg count was <5 epg.

**Table 1:** Comparative results of Allplex™ GI-Helminth(I) Assay and microscopy (*N* = 110 samples).

Targets	Number of samples with				Concordance: % (CI95)	Cohen’s kappa: *κ* (CI95)
	Micro +	Micro −	Micro +	Micro −		
	GIPH +	GIPH −	GIPH −	GIPH +		
Hookworms^a^	6	97	7	0	94% (87; 97)	0.60 (0.34; 0.86)
* A. duodenale*	2	108	0	0	100% (97; 100)	1.00 (1.00; 1.00)
* N. americanus*	4	104	2	0	98% (94; 100)	0.79 (0.51; 1.00)
*Ascaris* spp.	7	102	1	0	99% (95; 100)	0.93 (0.79; 1.00)
*E. vermicularis*	7	99	4	0	96% (92; 99)	0.76 (0.53; 0.98)
*Hymenolepis* spp.	20	87	1	2	97% (91; 99)	0.91 (0.82; 1.00)
*Strongyloides* spp.	28	73	7	2	92% (85; 96)	0.80 (0.68; 0.93)
*Taenia* spp.	5	105	0	0	100% (97; 100)	1.00 (1.00; 1.00)
*T. trichiura*	1	99	10	0	91% (84; 96)	0.15 (−0.11; 0.42)
All targets	67	10	30^b^	4^b^	70% (61; 78)	0.24 (0.08; 0.40)

Micro: Microscopy; GIPH: Allplex™ GI – Helminth(I) assay.

a As egg morphology does not allow species-level identification of hookworms, this row combines the results for both *A. duodenale* and *N. americanus* PCRs. The rows below consider only samples with positive culture, which allowed species-level identification.

b One sample was both “Microscopy +/GIPH −” for *A. lumbricoides* and “Microscopy −/GIPH +” for *S. stercoralis*, and was therefore counted in each column but considered as a single sample for the calculations.

**Table 2:** Performance of Allplex™ GI-Helminth(I) Assay for each target with or without bead-beating of the FecalSwab™ suspension.

Targets	Sensitivity compared to microscopy: % (*n*/*N*)	
	Without pretreatment	With pretreatment*
Hookworms	46% (6/13)	46% (6/13)
*Ascaris* spp.	88% (7/8)	100% (8/8)
*E. vermicularis*	64% (7/11)	73% (8/11)
*Hymenolepis* spp.	95% (20/21)	100% (21/21)
*Strongyloides* spp.	80% (28/35)	86% (30/35)
*Taenia* spp.	100% (5/5)	100% (5/5)
*T. trichiura*	9% (1/11)	91% (10/11)
All targets	70% (70/100)	84% (84/100)

* Only false-negative results without any pretreatment were re-extracted with prior bead-beating and re-amplified.

Considering all targets together, the molecular assay and microscopy showed fair agreement (*κ* between 0.21 and 0.40, 69% concordance), and this imperfect agreement was mainly caused by falsely negative results.

**Figure 1:**
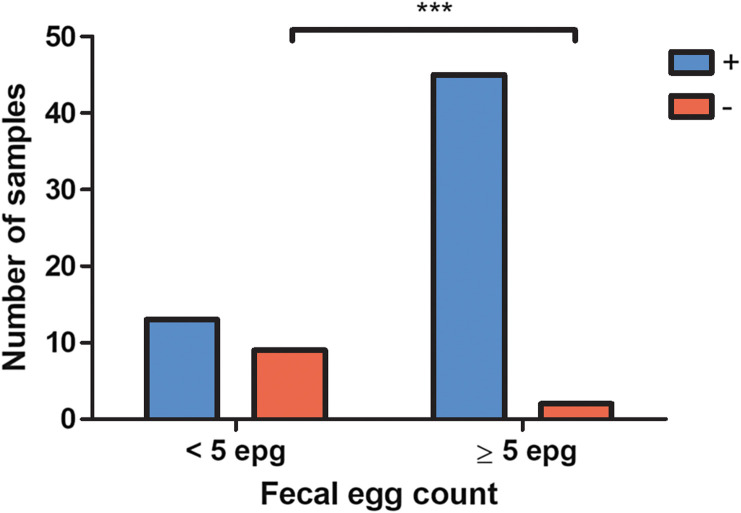
Proportion of samples detected with the Allplex™ GI-Helminth(I) Assay according to fecal egg count. Comparison of groups by Fischer’s exact test, *p* < 0.001.

As detailed above, the samples with a false-negative result from PCR were processed again with a previous bead-beating step of the FecalSwab™ suspension. Mechanical lysis improved the performance of the *T. trichiura* PCR detection, as only one sample with <5 epg remained negative (Table 2). However, this improvement was not observed for all targets, as all stool samples with hookworm eggs remained negative, and only 2 stool samples with *S. stercoralis* larvae became positive. For other targets, nearly all the false-negative results became positive when applying the bead-beating procedure. When applying this pretreatment, most of the remaining false-negative results of the Allplex™ GIPH assay were due to critically low parasitic loads (Fig. 1), as 41% of samples (9/22) with <5 epg were falsely negative, versus 4% (2/47) with ≥5 epg (*p* < 0.001).

**Figure 2:**
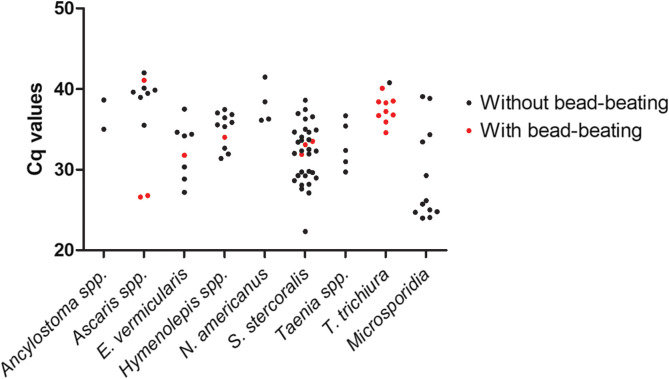
Cq values obtained with the Allplex™ GI-Helminth(I) Assay performed directly on stool samples (black) or after bead-beating (red).

The *C*_*q*_ values obtained with the Allplex™ GIPH assay also depended on the target (Fig. 2). However, signal was relatively weak for *Ascaris* spp., hookworms, and *T. trichiura*, for which *C*_*q*_ values were over 35. Of note, 5 samples tested with mechanical lysis, because falsely negative for a target, were otherwise positive for another target without bead-beating. Three of them were simultaneously positive for *S. stercoralis*, and mechanical lysis did not improve their *C*_*q*_, which reached 32.3, 33.4 and 33.7, versus 31.9, 33.5 and 33.1, without and with bead-beating, respectively. By contrast, the remaining two samples were also positive for *Ascaris* spp. eggs, and bead-beating dramatically improved the *C*_*q*_ (*C*_*q*_ of 26.6 and 26.8, respectively), compared to standard procedure (*C*_*q*_ of 42.0 and 40.1, respectively).

### Allplex™ GI-Helminth(I) Assay versus in-house microsporidia PCR

The collection included 10 stool samples previously tested positive by in-house PCRs; 9 contained *E. bieneusi* and 1 *E. intestinalis*. All ten were positive with the Allplex™ GIPH. As microsporidia PCR is not performed for all patients, but only those with immune deficiency and diarrhea, concordance and agreement could not be calculated. However, among the 100 remaining stool samples, one was found positive with the Allplex™ GIPH assay. This sample was confirmed with the in-house *E. bieneusi* PCR as a true positive of the multiplex PCR.

### *Schistosoma* in-house PCR

As *Schistosoma* spp. are not included in the Allplex™ GIPH panel, we wanted to verify whether the DNA extract was suitable for other single in-house PCRs to complement the diagnosis. Seventeen stool samples known to be positive for *S. mansoni* eggs by microscopy were extracted using the MICROLAB^®^ STARlet device, and an in-house *S. mansoni* PCR was performed on DNA extracts. The fecal egg counts for these samples ranged from <5 epg to 15–24 epg. All were positive with *C*_*q*_ ranging from 17.7 to 35.0. However, in one case, amplification was obtained only with the 1:10 dilution of the DNA extract and not with pure extract, indicating the presence of residual PCR inhibitors.

## Discussion

This study provides the first data on the performance of the Allplex™ GI-Helminth(I) Assay, by comparison to microscopy for helminth diagnosis, and to qPCR for microsporidia diagnosis. Results were heterogeneous and the performance depended on the target. Especially, some false-negative results were observed for hookworms and *S. stercoralis* detection, and almost all stool samples that contained *T. trichiura* eggs were negative with the Allplex™ GIPH assay. For the other targets, namely *Ascaris* spp., *E. vermicularis*, *Hymenolepis* spp., *Taenia* spp. and the microsporidia, the assay showed good results, as 84% (49/55) of the samples were positive by PCR. The performances were better when applying a bead-beating step before processing on the MICROLAB^®^ STARlet device. Particularly, this mechanical lysis enabled recovery of all except one false-negative result for *T. trichiura*. However, hookworm detection was not improved by bead-beating, and *S. stercoralis* detection was only slightly improved. If needed, the panel can be complemented by in-house PCRs using the same DNA extract, as we showed for the *S. mansoni* PCR.

Molecular methods for helminth diagnosis are still at their beginning. Although it seems disappointing not to detect all samples positive by microscopy, it should be kept in mind that such a performance is rarely observed. Importantly, previous studies on PCR performances for helminth diagnosis, even if carefully designed or associated with optimized extraction methods, frequently missed a few samples which tested positive by microscopy. Compared to the amount (1 g) of stool sample used for concentration methods, only 200–400 μL of diluted stool is usually used for DNA extraction, which could explain discrepant results. Another pitfall is the thickness of the egg wall which can be difficult to disrupt, and the frequent presence of PCR inhibitors in DNA extracts from stool samples. With the Allplex™ GIPH assay, the main challenge is the use of the same extraction method for all parasites targeted and the small sample volume 200 μL.

For each parasite targeted, we compared our results to those in the literature (Table 3) [3, 5, 10, 13, 15, 17, 18, 20, 25]. Overall, the performances of the Allplex™ GIPH assay were similar to other PCR techniques, except for hookworm and *T. trichiura* detection, the latter being improved when applying bead-beating pretreatment. The rates of false-negative results for *S. stercoralis* detection were variable, as they ranged from 12% to 50%, but this was mainly reported in old studies using *S. stercoralis* PCRs with poor performances [5, 6, 25]. It should be noted that in many comparisons of PCR to microscopy, egg detection relied on the Kato-Katz method [3, 5, 13, 15]. In our study, eggs were mostly detected using the merthiolate-iodine-formalin concentration method, which is known to be more sensitive as it uses a higher amount of sample [12]. This implies that the PCR performances observed would, in fact, probably be higher if compared to the Kato-Katz method. Finally, in most studies, as well as in the present work, helminth PCRs were evaluated as part of the initial diagnosis. As residual DNA could possibly be found in stool samples following treatment, further studies will be needed to assess whether PCR could be used for follow-up purposes, and as such, what timescale after treatment should be applied before re-sampling.

**Table 3:** Review of studies comparing the performances of PCR and microscopy for helminth detection.

Targets	Micro +	Micro +	Proportion of false-negative from PCR	Reference
	PCR +	PCR −		
*Ascaris* spp.	7	1	13%	This study
	154	7	4%	[17]
	34^a^	2^a^	6%^a^	[13]
	23	5	18%	[20]
	8	0	0%	This study (+bb)
	35^a^	1^a^	3%^a^	[13] (+bb)
	192	27	12%	[15] (+bb)
				
*E. vermicularis*^b^	7	4	36%	This study
	8	3	27%	This study (+bb)
				
Hookworms	6	7	54%	This study
	89	9	9%	[17]
	48	1	2%	[10]
	136	15	10%	[15] (+bb)
				
*Hymenolepis* spp.^b^	20	1	5%	This study
	21	0	0%	This study (+bb)
				
*Strongyloides* spp.^c^	28	7	20%	This study
	30	5	14%	This study (+bb)
	33	21	39%	[25]
	88	12	12%	[17]
	14	14	50%	[5]
				
*Taenia* spp.	5	0	0%	This study
	7	0	0%	[18]
				
*T. trichiura*	1	10	91%	This study
	18^a^	9^a^	33%^a^	[13]
	10	1	9%	This study (+bb)
	26^a^	1^a^	4%^a^	[13] (+bb)
	297	23	7%	[3] (+bb)

Micro: Microscopy; (+bb): with bead-beating pretreatment.

a In this study, different conditions were evaluated for sample preservation and pretreatment. In order to be in conditions similar to our study, the results shown in this table correspond to frozen samples without preservative.

b No published evaluation compared PCR and microscopy for *E. vermicularis* and *Hymenolepis* spp.

c As many evaluations of *S. stercoralis* have been published, sometimes with substantial bias, we focused on studies comparing real-time PCR targeting the 18S rRNA gene to a combination of parasitological methods as the reference, and without age restriction of the population.

The human hookworms, *Ancylostoma* spp. and *N. americanus*, were the targets of the Allplex™ GIPH assay with the poorest and not improvable performances. Of note, all the false-negative results for hookworms were observed for stool samples with rare eggs quantified by microscopic examination. The fact that mechanical lysis did not improve the performances suggests that the weakness of the assay is probably due to the amplification step. This is emphasized by a recent study which compared helminth DNA extraction with and without bead-beating, based on the analysis by numerous group-, genus- or species-specific PCRs [11]. This work showed a significant improvement of both in-house and commercial *N. americanus* PCRs using the bead-beating pretreatment. This was also supported by the high *C*_*q*_ values of the positive samples, ranging from 35 to 41.5, even for specimens containing numerous eggs or larvae. However, as species identification could not be performed for 5 out of 13 samples due to negative Harada-Mori filter paper culture, the eggs could correspond to helminths other than hookworms. For example, even though these parasites are rare in humans, eggs of *Trichostrongylus* spp. or *Oesophagostomum* spp. can easily be misidentified as hookworm eggs. Interestingly, as specified above, one stool sample that contained hookworm eggs was positive for the *Strongyloides* spp. PCR but the *Ancylostoma* spp. and *N. americanus* PCRs both tested negative. This could be explained if the patient was in fact infected with *Strongyloides fuelleborni* (as *S. stercoralis* eggs are not shed with stools), and if eggs were misidentified as hookworm eggs. However, this putative explanation is quite unlikely as *S. fuelleborni* eggs are rather embryonated compared to hookworm eggs.

PCR targeting *E. vermicularis* and *S. stercoralis* showed moderate performances compared to microscopy, as they yielded 36% (4/11) and 20% (7/35) false-negative results, respectively. However, stool examination is known not to be the most sensitive method for the diagnosis of these parasites. As the *E. vermicularis* females do not lay eggs in the intestinal lumen, the best diagnostic method is the scotch tape test, and, for *S. stercoralis*, serological techniques are known to be much more sensitive than stool examination [7]. Moreover, we know that preservation of DNA by storage at −80 °C without preservative is of limited efficacy. In a previous study, we observed better performances in a prospective evaluation using fresh stool samples, than in a retrospective evaluation using frozen samples from our bank [2]. Therefore, further prospective studies would be welcome to confirm the performances of the Allplex™ GIPH on fresh samples.

This kind of molecular assay raises thoughts about its place in the diagnostic strategy. In endemic countries, the cost and logistic aspects of such multiplex PCR is not designed for primary or secondary care medical centers, but could be suitable for tertiary care hospitals, even though it does not allow the detection of several helminths to which these populations are exposed. Hence, microscopic examination of stool samples will probably remain the gold standard due to its cost-effectiveness and efficiency, provided that it is performed by skilled and trained operators to achieve good sensitivity. However, this multiplex molecular assay could be implemented in tertiary centers with human and financial resources. We previously showed that the FecalSwab™ allows stool preservation at +4–8 °C until one week before DNA extraction and amplification [2], which allows strategies consisting of sample collection in the field, then transportation and analysis at the reference center.

In countries less exposed to helminthiasis (e.g. European countries), the picture is quite the opposite. An increasing proportion of private clinical laboratories and secondary care hospitals have a molecular biology platform, allowing them to implement these types of multiplex panels. In this perspective, the Allplex™ assay could be an easy tool to diagnose most common helminths with fair performances, except for migrants who might be infected with helminths not targeted by the assay. By contrast, reference centers need to maintain specialized expertise to diagnose a broad spectrum of diseases, and thus cannot abandon classical parasitological methods.

Overall, the Allplex™ GIPH assay could be a useful tool to detect numerous parasites in a same run, but suffers from a limited number of parasitic targets. The choice of combining microsporidia detection to a panel of helminths seems inappropriate, as the detection of these fungi is usually indicated in immunocompromized patients, whereas the helminths panel is useful for screening migrants or travelers. Additionally, the incorporation of *Schistosoma* spp. into the panel of targets would be welcome, as these parasites are major pathogens worldwide. Improvement of the extraction process by addition of mechanical lysis is necessary to reach an equivalent sensitivity to that of microscopy, but the sensitivity of hookworm detection remains poor. Of interest, the DNA extracts obtained from the MICROLAB^®^ STARlet device are suitable for complementary in-house PCR. Overall, our study showed that the Allplex™ GI-Helminth(I) Assay needs to be improved before being used for routine patient management.

## Conflict of interest

The PCR reagents were purchased from the Seegene Company at reduced price, but the firm did not take part in the writing of the manuscript, nor its submission.
